# Clinical characteristics and outcome in patients with ST-segment and non-ST-segment elevation myocardial infarction without obstructive coronary artery: an observation study from Chinese population

**DOI:** 10.1186/s12872-021-02359-x

**Published:** 2022-01-29

**Authors:** Menghuan Li, Yuan He, Iokfai Cheang, Zhiyong Zhang, Yang Liu, Hui Wang, Xiangqing Kong

**Affiliations:** 1 Department of Cardiology, The First Affiliated Hospital of Nanjing Medical University, Guangzhou Road 300, Nanjing, 210029 China; 2 Department of Cardiology, Suqian First People’s Hospital of Suqian City, Suqian, China; 3 Gusu School, Nanjing Medical University, Suzhou, China

**Keywords:** Acute myocardial infarction (AMI), Myocardial infarction without obstructive coronary artery (MINOCA), Outcome, Clinical characteristics, Cardioprotective therapy

## Abstract

**Background:**

The disparity between ST-segment and non-ST-segment elevation myocardial infarction without obstructive coronary artery (STE-MINOCA and NSTE-MINOCA) are unclear. Our study aims to compare the clinical features and outcomes in patients with STE-MINOCA and NSTE-MINOCA.

**Methods:**

This cross-sectional study consecutively enrolled patients diagnosed with acute myocardial infarction (AMI) from January 2013 to January 2020. MINOCA were identified as angiographic stenosis < 50%. Clinical characteristics, angiographic features, and clinical outcomes of STE-MINOCA and NSTE-MINCOA were documented. The primary endpoint was composite events in the different time periods.

**Results:**

A total of 1966 AMI patients were screened, 107 (5.4%) were diagnosed as MINOCA. Among, there were 34 (31.8%) of STE-MINOCA and 73 (68.2%) of NSTE-MINOCA. STE-MINOCA group were younger, had lower N-terminal pro-brain natriuretic peptide (NT-proBNP), and smaller left atrial diameter (*P* < 0.05). Dual antiplatelet therapy (DAPT) was more likely to be prescribed to STE-MINOCA patients (*P* = 0.015). During median follow-up time of 24.5 months, STE-MINOCA group also demonstrated lower risks for primary endpoint and cardiovascular-related (CVS) rehospitalization. In univariate Cox regression analyses, NSTE-MINOCA showed an increased risk of long-term primary endpoint (HR 2.57, 95 CI%: 1.10–6.02) and CVS-related rehospitalization (HR 3.14, 95% CI: 1.16–8.48). After adjusting for NT-proBNP and DAPT, NSTE-MINOCA remained an independent risk factor for CVS-related rehospitalization in long-term follow-up (HR 2.78, 95% CI: 1.03–7.49).

**Conclusion:**

Although STE-MINOCA and NSTE-MINOCA patients showed similar clinical characteristics, NSTE-MINOCA group presented a worse long-term outcome mainly driven by CVS-related hospitalization which suggested that NSTE-MINOCA patients might also require prompt medical attention.

## Introduction

Myocardial infarction without obstructive coronary artery (MINOCA) is a distinctive entity differing from classic acute myocardial infarction (AMI) [1]. Previous literature reported a prevalence of MINOCA of 5%-15% [2–5]. AMI can be classified as ST-segment elevation myocardial infarction (STEMI) and non-ST-segment elevation myocardial infarction (NSTEMI) according to the presentation of electrocardiogram (ECG) [6]. Generally, the main pathogenesis of classic AMI is the plaque disruption or erosion based on significant stenosis, subsequently forming a cascade thrombosis [7]. Likewise, MINOCA can also be categorized by ST-segments changes (STE-MINOCA and NSTE-MINOCA).

However, the mechanism of MINOCA was poorly understood. Several specific causes were proposed, including atherosclerotic causes (plaque rupture, plaque erosion) and non-atherosclerotic causes (epicardial coronary spasm, coronary microvascular dysfunction, coronary embolism, spontaneous coronary artery dissection, and supply–demand mismatch) [1]. For MINOCA, any causes leading to total occlusion of a coronary artery tend to have STEMI, or partial occlusion of arteries tend to have NSTEMI [7]. Reportedly, worse short-term outcome was observed in STEMI patients, and worse long-term outcome in NSTEMI patients [8, 9]. Consider the distinct pathophysiology in MINOCA, there were lack of evidence regarding the outcomes in patients with STE-MINOCA and NSTE-MINOCA.

Therefore, the purpose of our study was to investigate the characteristics and prognosis between STE-MINOCA and NSTE-MINOCA patients in Chinese population.

## Methods

### Definition

According to the “Fourth Universal Definition of Myocardial Infarction” criteria [6] acute myocardial infarction (AMI) was defined as follows: (1) detection of a rise or fall of cardiac troponin (cTn) with at least one value exceeding the 99th percentile upper reference limit. (2) clinical evidence of infarction evidenced by at least one of the following: a) symptoms of myocardial infarction. b) new ischemic electrocardiographic changes. c) development of pathological Q waves. d) imaging evidence of new loss of viable myocardium or new regional wall motion abnormality. e) identification of a coronary thrombus by angiography. Nonobstructive coronary arteries on angiography were defined as any major epicardial vessels within 50% stenosis including normal coronary arteries (no angiographic stenosis), mild luminal irregularities (angiographic disease < 30% stenosis), and moderate coronary atherosclerotic lesions (stenosis > 30% but < 50%).

Thus, MINOCA was diagnosed as myocardial infarction with nonobstructive coronary artery stenosis and no specific alternate causes for above clinical presentations. In accordance with ST segments changes on electrocardiogram (ECG), MINOCA was classified as STE-MINOCA and NSTE-MINOCA.

To further characterize the pathological changes, the following features were assessed accordingly. Coronary thrombosis was identified as ground glass opacification or filling defect by coronary angiography, or detected by optical coherence tomography (OCT). Plaque disruption were determined by OCT as discontinuous cap of lipid plaque. Coronary spasm was considered as transient stenosis of angiographic artery or absence of stenosis after intracoronary administration of nitrates. Coronary slow flow was confirmed by corrected TIMI frame count.

### Study population

**Fig. 1:**
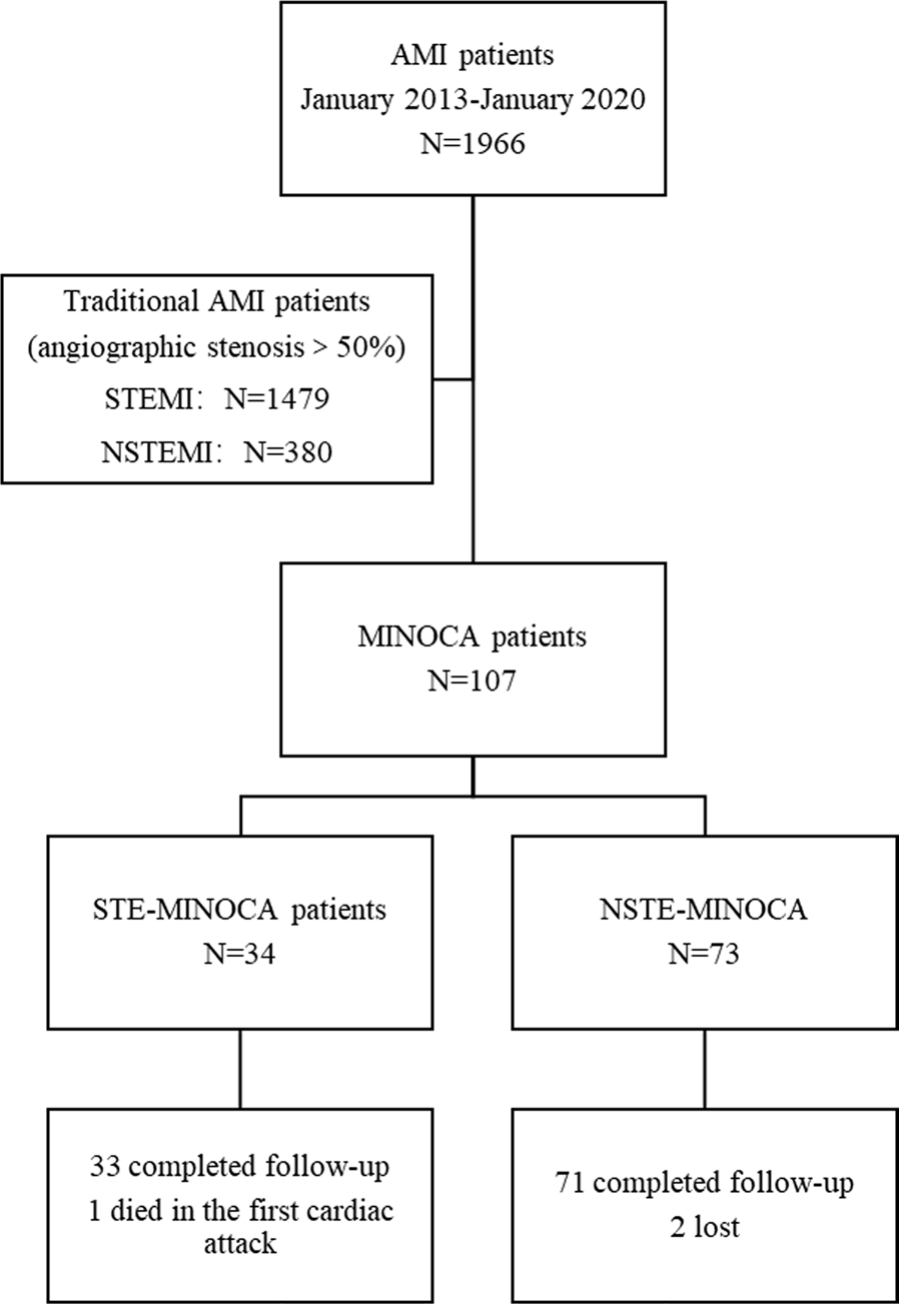
Study flow chart

This is a cross-sectional study comprised of all consecutive patients with MINOCA in the First Affiliated Hospital of Nanjing Medical University from January 2013 to January 2020. Patients with conformed diagnosis of AMI according to guidelines [6] were eligible for this study. Among 1996 patients with AMI, 107 participants were diagnosed as MINOCA. Patients were further assigned to different group according to the presentation of ST-segments. Of those, 73 patients (68.2%) were diagnosed as NSTE-MINOCA, 34 patients (31.8%) were diagnosed as STE-MINOCA (Fig. 1).

Exclusion criteria were thrombolytic therapy prior to coronary angiography, women at pregnant or breastfeeding stage, presumed myocarditis, takotsubo syndrome, an expected survival time of less than one year due to any malignancy. Our study was approved by the independent ethical review board of the First Affiliated Hospital of Nanjing Medical University and in accordance with the Declaration of Helsinki. Written informed consents were obtained from all participants.

### Data collection

Baseline characteristics including demographics, vitals at admission, medical history, and medication were collected. Blood samples were collected to assess high-sensitive cardiac troponin T (hs-cTnT) at the peak, total cholesterol (TC), triglyceride (TG), low-density lipoprotein cholesterol (LDL-C), high-density lipoprotein cholesterol (HDL-C), lipid protein a (Lp-a), alanine aminotransferase (ALT), aspartate aminotransferase (AST), uric acid (UA), serum creatinine (SCr), fasting blood glucose, glycate hemoglobin, N-terminal pro-brain natriuretic peptide (NT-proBNP), etc. The data of transthoracic echocardiography (TTE) and coronary angiography were documented.

In order to differentiate selected ambiguous cases of AMI, cardiac magnetic resonance imaging (CMRI) was used to exclude other potential causes, including myocarditis, takotsubo syndrome, and cardiomyopathies. Left ventriculography were performed to differentiate AMI from takotsubo syndrome. Intravascular ultrasound (IVUS) or OCT was applied to selective patients for insight etiology of plaque disruption or erosion.

### Endpoint and follow-up

The primary endpoint was composite events in different time period. The in-hospital composite events included cardiac death, malignant arrhythmia, and heart failure (HF). The 1-year or long-term composite events were defined as all-cause mortality, HF, recurrence of AMI (re-AMI), cardiovascular-related (CVS) rehospitalization and stroke. And the secondary endpoint was each component of the primary outcome.

The diagnosis of HF is established according to the ESC guidelines for the management of HF. Malignant arrhythmia referred to tachyarrhythmia and bradyarrhythmia that required drug or medical equipment support. Re-AMI was defined as typical chest pain with dynamic change of ST-segments or T wave change on the ECG, or elevation of serum cTn level. Stroke referred to ischemic cerebral infarction or hemorrhagic stroke detected by computed tomography or magnetic resonance.

Patients were followed up from discharge through outpatient visit, inpatient chart review, and/or telephone interview every six months until the end of follow-up. The endpoint events were confirmed by reviewing of medical records or contacting with patients, their family members, and/or physicians.

### Statistical analysis

Data distribution was assessed by Kolgormonov-Smirnov test. As cTnT, AST, NT-proBNP were tested as skewness distribution, therefore were presented as median with interquartile range (IQR) and compared by non-parametric Mann–Whitney U test. Numerical variables with normal distribution were presented as the mean ± standard deviation and were compared by independent sample student’s t-test. Categorical variables were calculated using counts and percentages and were evaluated by Chi-square test or Fisher exact test as appropriate.

The cumulative incidence was estimated by Kaplan–Meier (K-M) method and were compared using log-rank test. Cox proportional-hazards models were applied to calculate hazard ratios with and without adjustment. We included in the multivariable model only when variables showed *P* < 0.05 at univariate analysis. As NT-proBNP reflects the state of cardiac function, and dual antiplatelets therapy (DAPT) is the basic medication in patients with AMI. These variables were used as covariables in the model to identify independent risk factors for clinical endpoint in patients with MINOCA. A two-sided *P* < 0.05 was considered as statistical significance. All the analyses were performed using SPSS version 25 software.

## Results

### Baseline characteristics

Figure 1 showed the detailed flow chart of the study. Of the 1966 patients with AMI, 1859 (94.6%) having obstructive myocardial infarction: 1479 (79.6%) with STEMI and 380 (20.4%) with NSTEMI. There were 107 (5.4%) patients identified as MINOCA and median follow-up time was 24.5 (IQR:12.0–44.5) months.

Among the patients with MINOCA, 34 (31.8%) were categorized to STE-MINOCA and 73 (68.2%) were categorized to NSTE-MINOCA. The proportion of patients with NSTE-MINOCA was greater than that of patients with NSTEMI.

Table 1 showed the baseline clinical characteristics between two groups. Compared with patients with NSTE-MINOCA, patients with STE-MINOCA were younger (57.09 ± 13.73 vs 62.55 ± 11.80, *P* = 0.037), had lower NT-proBNP level (310[88–708] vs 575[143–1450], *P* = 0.038), and smaller left atrial diameter (LAD) (35.55 ± 3.68 vs 37.94 ± 5.25, *P* = 0.021). Gender, past medical history, other myocardial biomarkers, serum lipid profile, and TTE parameter were similar between groups (All *P* > 0.05).

**Table 1:** Baseline characteristics

Characteristics	STE-MINOCA (N = 34)	NSTE-MINOCA (N = 73)	*P*
Demographics			
Age (years)	57.09 ± 13.73	62.55 ± 11.80	0.037
Women, n(%)	9(26.5)	25(32.9)	0.504
Vital signs			
Systolic blood pressure	127.09 ± 20.82	131.04 ± 18.75	0.329
Diastolic blood pressure	78.76 ± 13.78	75.95 ± 10.72	0.251
Risk factors			
Hypertension, n(%)	23(67.5)	42(57.5)	0.319
Diabetes mellitus, n(%)	7(20.6)	11(15.1)	0.477
Atrial fibrillation, n(%)	3(8.8)	7(9.6)	1.000
Stroke, n(%)	3(8.8)	6(8.2)	1.000
COPD, n(%)	0(0)	2(2.7)	1.000
Smoking, n(%)	12(35.3)	33(45.2)	0.334
Alcohol, n(%)	7(20.6)	18(24.7)	0.643
Biochemical indicators			
cTnT (ng/L)	635 [145–2001]	410 [181–1024]	0.438
TC (mmol/L)	4.09 ± 1.30	3.97 ± 0.94	0.588
TG (mmol/L)	1.60 ± 0.59	1.37 ± 0.64	0.091
HDL-C (mmol/L)	1.09 ± 0.38	1.11 ± 0.33	0.800
LDL-C (mmol/L)	2.38 ± 0.94	2.38 ± 0.74	0.997
RC (mmol/L)	0.59 ± 0.60	0.48 ± 0.34	0.370
AST (U/L)	47.40 [26.93–105.33]	39.80 [23.58–66.50]	0.128
Uric acid (umol/L)	322.49 ± 73.73	343.73 ± 94.50	0.269
Serum creatine (mmol/L)	75.44 ± 29.89	72.99 ± 21.06	0.640
Fasting blood glucose (mmol/L)	5.36 ± 1.35	5.34 ± 1.12	0.950
HbA1c (%)	5.42 ± 0.87	5.44 ± 1.12	0.514
NT-proBNP (pg/ml)	310 (88–708)	575 (143–1450)	0.038
Echocardiogram parameters			
LVEF(%)	59.98 ± 8.17	60.58 ± 9.01	0.743
EF < 50%	4 (11.8)	7 (9.6)	0.740
LAD(mm)	35.55 ± 3.68	37.94 ± 5.25	0.021
LVDs(mm)	32.09 ± 4.22	33.12 ± 7.14	0.448
LVDd(mm)	48.48 ± 5.43	49.24 ± 7.11	0.594
FS(%)	33.09 ± 3.51	33.26 ± 5.59	0.870
Wall motion abnormality, n(%)	10 (29.4)	15 (20.5)	0.313

The numbers in round brackets represent percentages and those in square brackets represent IQR. The rest are presented as mean ± SD

*COPD* chronic obstructive pulmonary disease; *cTnT* cardiac troponin T; *TC* total cholesterol; *TG* triglyceride; *LDL-C* low-density cholesterol; *HDL-C* high-density cholesterol; *RC* residual cholesterol; *ALT* alanine aminotransferase; *AST* aspartate aminotransferase; *HbA1C* hemoglobin A1C; *NT-proBNP* N-terminal pro-brain natriuretic peptide; *LVEF* left ventricular ejection fraction; *LAD* left artery diameter; *LVDs* left ventricular diameter of end-systole; *LVDd* left ventricular diameter of end-diastole; *FS* fractional shortening

### Angiographic findings

Table 2 presented the angiographic features of patients with MINOCA. Approximately half of vessels showed normal or near normal artery in MINOCA patients (28 normal coronary artery and 24 minimal lumen irregularities) and proportion of angiographic severity had no significant difference in two groups. For the vessels with atherosclerotic plaque, similar proportion of single vessel lesions, two-vessels lesions, and three-vessels lesions were observed between groups.

**Table 2:** Angiographic characteristics between two groups

Characteristics	All (N = 107)	STE-MINOCA (N = 34)	NSTE-MINOCA (N = 73)	*P*
Angiographic stenosis				0.287
Normal coronary arteries	28 (26.2)	10 (29.4)	18 (24.7)	
Minimal lumen irregularities	26 (24.3)	5 (14.7)	21 (28.8)	
Mild to moderate stenosis	53 (49.5)	19 (55.9)	34 (49.3)	
Vessels with any stenosis				0.597
1-vessel disease	28 (35.4)	13 (54.2)	25 (45.5)	
2-vessles disease	22 (27.8)	7 (29.2)	15 (27.3)	
3-vessles disease	19 (24.1)	4 (16.7)	15 (27.3)	
Other findings				
Coronary thrombosis	3 (2.8)	1 (2.9)	2 (2.7)	1.000
Plaque disruption	2 (1.9)	1 (2.9)	1 (1.4)	0.539
Coronary spasm	10 (9.3)	4 (11.8)	6 (8.2)	0.723
Coronary slow flow	18 (16.8)	6 (17.6)	12 (16.4)	1.000
Special inspection				
Left ventriculography	5 (4.7)	1 (2.9)	4 (5.5)	1.000
OCT	3 (2.8)	2 (5.9)	1 (1.4)	0.236

*OCT* optical coherence tomography

The possible mechanism of MINOCA was identified as 3 (2.8%) for coronary thrombosis, 2 (1.9%) for plaque disruption, 10 (9.3%) for coronary spasm, and 18 (16.8%) for coronary slow-flow. Further evaluation including left ventriculography and OCT were rarely performed, only 5(4.7%) patients underwent left ventriculography, 3(2.8%) patients received OCT examination.

### Medication at discharge

**Fig. 2:**
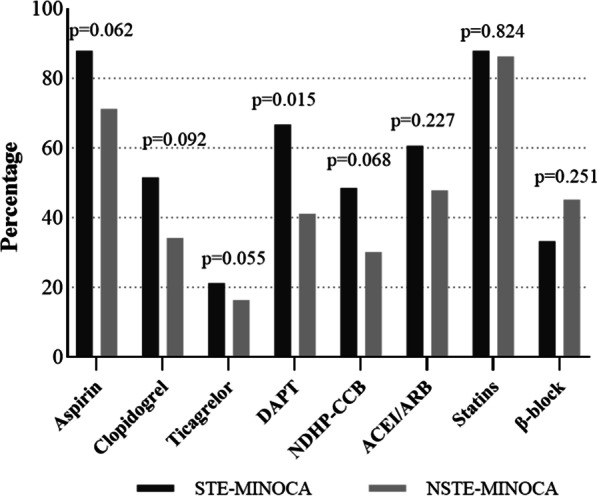
Use of cardioprotective medications between groups

Medication regimen at discharge between groups were demonstrated in Fig. 2. Dual antiplatelet therapy (DAPT) consists of aspirin and P2Y12 receptor inhibitor. Aspirin, clopidogrel, and ticagrelor were more used in patients with STEMI numerically (*P* > 0.05). However, patients with NSTE-MINOCA were less likely to be prescribed DAPT on discharge (41.4% vs 66.7%, *P* = 0.015). There were no significant differences between groups on angiotensin-converting enzyme inhibitors or angiotensin receptor blockers (ACEI/ARB) (60.6% vs 47.9%, *P* = 0.227), non-dihydropyridine calcium channel blockers (NDHP-CCB) (48.5% vs 30.1%, *P* = 0.068), statins (87.9% vs 86.3%, *P* = 0.824) and beta receptor blockers (33.3% vs 45.2%, *P* = 0.251) (Fig. 2).

### Clinical outcome between STE-MINOCA and NSTE-MINOCA

A total of 6 patients had in-hospital primary endpoint, including 1 (2.9%) for cardiac death, 3 (2.8%) for malignant arrhythmia, and 2 (1.9%) for acute HF. No significant difference was found between two groups.

In terms of 1-year outcome, patients with NSTE-MINOCA had higher rate of CVS-related rehospitalization (17.8% vs 2.9%, *P* = 0.035), but no significant difference in all-cause mortality, re-AMI, HF, and stroke compared with patients with STE-MINOCA.

There were 34 (31.8%) cases of primary endpoint occurring during the long-term follow-up in MINOCA patients, including 2 (1.9%) of all-cause mortality, 2 (1.9%) of re-AMI, 2 (1.9%) of HF, 3(2.8%) of stroke, and 27(25.2%) of CVS-related rehospitalization. There were no statistical differences in terms of long-term outcome between patients with STE-MINCOA and NSTE-MINOCA (Table 3).

**Table 3:** Primary and secondary endpoint rate between STE-MINOCA and NSTE-MINOCA

	All (N = 107)	STE-MINOCA (N = 34)	NSTE-MINOCA (N = 73)	*P* value
In-hospital				
Composite events	6 (5.6)	1 (2.9)	5 (6.8)	0.662
Cardiac death	1 (0.9)	1 (2.9)	0 (0)	0.318
Malignant arrhythmia	3 (2.8)	0 (0)	3 (4.1)	0.555
Heart failure	2 (1.9)	0 (0)	2 (2.7)	0.562
1 year				
Composite events	19 (17.9)	3 (9.1)	16 (21.9)	0.171
All cause of death	0 (0)	0 (0)	0 (0)	1.000
Re-AMI	2 (1.9)	2 (5.9)	0 (0)	0.099
Heart failure	2 (1.9)	0 (0)	2 (2.7)	0.562
Stroke	2 (1.9)	0 (0)	2(2.7)	0.562
CVS-related rehospitalization	14 (13.3)	1 (2.9)	13 (17.8)	0.035
Long-term				
Composite events	34 (31.8)	7 (20.6)	27 (37.0)	0.090
All cause of death	2 (1.9)	0 (0)	2 (2.7)	0.562
Re-AMI	2 (1.9)	2 (5.9)	0 (0)	0.099
Heart failure	2 (1.9)	0 (0)	2 (2.7)	0.562
Stroke	3 (2.8)	0 (0)	3 (4.1)	0.555
CVS-related rehospitalization	27 (25.2)	5 (14.7)	22 (30.1)	0.087

*AMI* acute myocardial Infraction; *CVS* cardiovascular

**Fig. 3:**
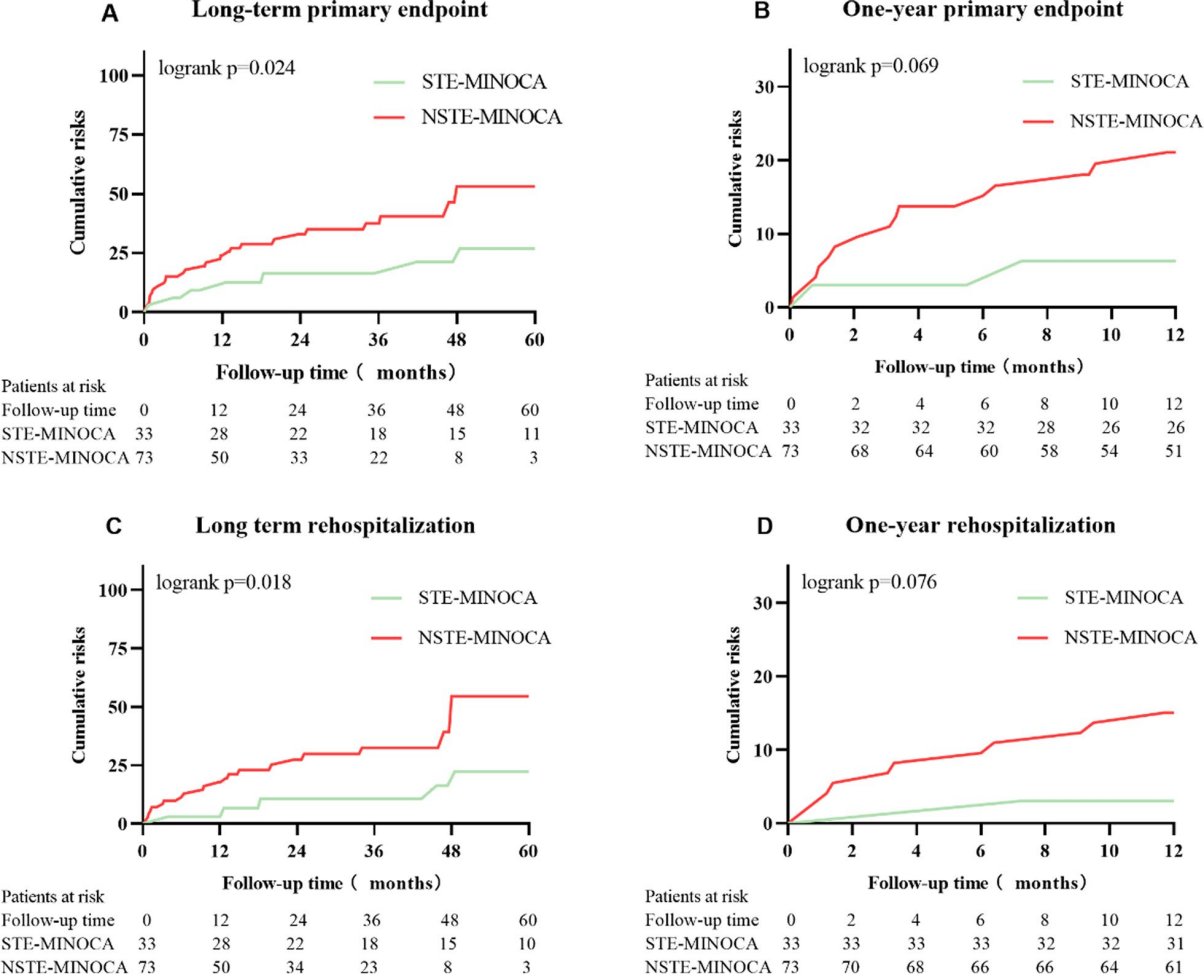
Kaplan–Meier survival curve of STE-MINOCA and NSTE-MINOCA during one year and long-term follow-up. **A** Cumulative long-term primary endpoint risks between STE-MNOCA and NSTE-MINOCA. **B** Cumulative one-year primary endpoint risks between STE-MINOCA and NSTE-MINOCA. **C** Cumulative long-term cardiovascular-related rehospitalization risks between STE-MINOCA and NSTE-MINOCA. **D** Cumulative one-year cardiovascular-related rehospitalization risks between STE-MINOCA and NSTE-MINOCA

Kaplan–Meier analysis showed that patients with NSTE-MINOCA had a trend of higher cumulative risks of composite endpoint and rehospitalization during the first year of follow-up, but log-rank test showed no statistical difference (Fig. 3). In addition, NSTE-MINOCA was statistically associated with higher risks for composite endpoint and CVS-related rehospitalization during long-term follow-up compared to STE-MINOCA (All *P* < 0.05).

In a univariate Cox regression analysis, NSTE-MINOCA had higher increased risks for long-term primary endpoint (HR 2.57, 95 CI% 1.10–6.02) and CVS-related rehospitalization (HR 3.14, 95% CI: 1.16–8.48)**.** In a multivariate analysis, adjusted for NT-proBNP and DAPT, NSTE-MINOCA retained a significantly higher hazard ratio for CVS-related rehospitalization in the long-term follow-up (HR 2.78, 95% CI: 1.03–7.49), but showed no significant difference in primary endpoint (HR 1.856, 95% CI:0.77–4.43) (Table 4).

**Table 4:** Unadjusted and adjusted risk for clinical outcome in NSTE-MINOCA versus STE-MINOCA

	Univariate analysis		Multivariate analysis	
	HR (95% CI)	*P*	HR (95% CI)	*P*
One year				
Primary endpoint	3.510 (0.803–15.352)	0.095	2.847 (0.603–13.442)	0.186
CVS-related rehospitalization	5.094 (0.658–39.455)	0.119	3.892 (0.487–31.089)	0.200
Long term				
Primary endpoint	2.570 (1.098–6.017)	0.030	1.846 (0.770–4.425)	0.169
CVS-related rehospitalization	3.136 (1.160–8.483)	0.024	2.778 (1.031–7.487)	0.043

*CVS* cardiovascular

## Discussion

This study compared the clinical and prognostic disparity between STE-MINOCA and NSTE-MINOCA. While age, NT-proBNP, and LAD were shown significant between STE-MINOCA and NSTE-MINOCA groups, other clinical characteristics showed no statistical differences. Patients with MINOCA often showed coronary arteries with mild atherosclerosis, but intracoronary imaging or coronary functional examination for further etiology were seldom performed. Patients with STE-MINOCA were more likely to be prescribed DAPT. There were no significant differences in in-hospital primary endpoint between groups. However, NSTE-MINOCA presented a trend of worse clinical outcome in terms of composite events and CVS-related rehospitalization in the longer-term follow-up.

Previous studies demonstrated that demographic and clinical characteristics were comparable between MINOCA and AMI-CAD [3–5, 10]. Patients with MINOCA are often younger, female, and less comorbidity. Moreover, presentation of NSTE-MINOCA account for great proportion in all MINOCA cases. COAPT study showed that NSTE-MINOCA patients were more common in MINOCA (STE-MINOCA vs NSTE-MINOCA, 73.7% vs. 26.3%) [11]. Our studies presented a prevalence of MINOCA of 5.4% in all AMI cases, and 68.2% NSTE-MINOCA cases among the MINOCA cases, which consisted with the previous studies [4, 10].

Clinical features in patients with STEMI and NSTEMI were well established [8, 9]. Patients with NSTEMI are usually older, women, higher incidence of hypertension, diabetes mellitus, dyslipidemia, and renal dysfunction. Consistently, our results showed that NSTE-MINOCA shared similar clinical features with NSTEMI as younger age and lower level of NT-proBNP. However, there were no significant differences on gender, lipid profiles, and past medical history between STE-MINOCA and NSTE-MINOCA.

Lower than 50% angiographic stenosis defined as non-obstructive is somewhat arbitrary but pragmatic in clinical practice [1]. Visual estimation of lesion severity is variable by different clinical physicians, it is useful to categorize MINOCA into three degrees according to angiographic diameter stenosis with absolute normal artery, near normal artery (0–30%) and mild-moderate stenosis (30–50%) [1]. Montone et al. reported 54.8% near normal vessels in MINOCA subjects [4]. COAPT study also demonstrated that 63.2% of MINOCA patients had no angiographic evidence of CAD [11]. In our study, normal or near normal vessels accounted for 50.5% in all cases, which was close to the results of previous reports.

Plaque disruption occurred at the segments that appeared angiographically normal in approximately half of the MINOCA cases with rupture and/or ulceration [14]. While large plaque of coronary artery was associated with poorer prognosis [13], these vessels with mild to moderate stenosis are recommended to perform functional coronary tests or intracoronary examinations to determine the specific etiology for MINOCA [1]. However, these specific intracoronary inspections such as IVUS or OCT were insufficiently performed to patients with MINOCA [1]. Previous studies reported that OCT or IVUS was seldom performed due to its high financial burden [1, 4]. Likewise, although only three patients underwent OCT examination in our study, all patients have positive findings, of which, 2 were identified as plaque disruption and 1 was recognized as coronary white-thrombosis attached to vessel wall. For patients with MINOCA, intracoronary examinations are crucial to seek underlying mechanisms and to guide the optimal management.

For traditional AMI patients, secondary prevention medications (includes DAPT, statins, ACEI/ARB, and β-blockers) are strongly recommended [7]. On the contract, application of the secondary prevention therapy to patients with MINOCA remain controversial. A study from SWEDEHEART registry reported that DAPT might not be beneficial for patients with MINOCA [15]. Paolisso et al. showed that the use of DAPT and β-blockers were not associated with long-term prognosis [16]. However, MINOCA patients with explicit plaque disruption should be considered to receive anti-platelet agents [15]. As mentioned above, 38% of MINOCA patients had coronary plaque disruption found by OCT. One small study demonstrated that patients of ACS with plaque erosion receiving dual anti-platelet agents alone without stenting showed an acceptable outcome [17], which meant, to some extent, DAPT may have beneficial effects to patients with coronary plaque disruption. This emphasizes that intracoronary imaging is conducive to guide medication use and to optimize these patients’ prognosis.

We observed that STE-MINOCA patients were more likely to be discharged with aspirin or DAPT than NSTE-MINOCA in our study. Due to lack of guidelines of MINOCA managements, decision of anti-platelet therapy is challenging [16]. Thus, adopting anti-platelet therapies to STE-MINOCA patients was more crucial and easier to than NSTE-MINOCA.

Approximately 25% of patients with MINOCA will experience recurrent angina in the subsequent one year affecting patients’ quality of life [19]. In terms of clinical outcomes, previous studies indicated that the in-hospital mortality rate of MINOCA was quite low. While our study observed only 1 (0.9%) in-hospital death in 107 patients, ACTION-GWTG study showed similar in-hospital death rate of 1.1% in 19,000 patients with MINOCA. While our study observed only 1 (0.9%) in-hospital death in 107 patients, However, a meta-analysis demonstrated that 1-year mortality rate was risen up to 4.7%. Furthermore, SWEDEHEART study showed the long-term mortality rate of 13.4%, re-AMI of 7.1% and rehospitalization of 10%. And our results presented long term mortality rate of 2.9% and rehospitalization rate of 25.2%. It was reported that these substantial recurrence events were more frequent in MINOCA patients than in general population [1].

A study of Danish cohort, NSTEMI patients showed poorer outcome in multivariate analysis [8]. Spanish cohort study presented that higher 7-year survival rate was observed in the cohort of NSTEMI compared with the cohort of STEMI [18]. However, Hyun-Woong et al. reported worse early clinical outcome in patient with STEMI and more late-term favorable outcome in patients with NSTEMI [9]. As it shown in our study, STE-MINOCA appeared to show better outcome than NSTE-MINOCA during long-term follow-up, whereas no significant difference in 1-year follow-up. In this sense, MINOCA was not benign, especially for NSTE-MINOCA. In conclusion, recognizing these different characteristic of various AMI types is curial for the optimal management. Intravascular assessment could provide important evidence in such aspect and should be considered in MINOCA patients.

There are several limitations in current study. Firstly, use of CMR is strongly encouraged to exclude alternative diagnoses, however CMR examinations were seldom performed in our study, which may influence the accuracy of diagnosis of MINOCA. Secondly, approximately half of patients had mild to moderate atherosclerosis in this study, further examination assisting differentiation such as fractional flow reserve (FFR) testing to explore functional significant stenoses were no included. Also, intracoronary imaging to identify the possible pathological changes of plaque disruption and/or erosion were seldomly perform. Additionally, retrospective design, single-center study and small size may contribute a bias in present study.

## Conclusion

Our study indicates that NSTE-MINOCA accounted for approximately two-thirds of MINOCA. Compared with STE-MINOCA, NSTE-MINOCA seemed to be associated with worse long-term clinical outcomes, which was mainly driven by CVS-related hospitalization.

## Data Availability

The data analyzed in this study are not publicly available due to the privacy policy of the hospital but are available from the corresponding author on reasonable request.

## Abbreviations

STE-MINOCA: ST-segment elevation myocardial infarction without obstructive coronary artery
NSTE-MINOCA: Non-ST-segment elevation myocardial infarction without obstructive coronary artery
AMI: Acute myocardial infarction
NT-proBNP: N-terminal pro-brain natriuretic peptide
DAPT: Dual antiplatelet therapy
CVS: Cardiovascular
HR: Hazard ratio
ECG: Electrocardiogram
OCT: Optical coherence tomography
hs-cTnT: High-sensitive cardiac troponin T
TC: Total cholesterol
TG: Triglyceride
LDL-C: Low-density cholesterol
HDL-C: High-density cholesterol
Lp-a: Lipid protein a
ALT: Alanine aminotransferase
AST: Aspartate aminotransferase
UA: Uric acid
SCr: Serum creatinine
CMRI: Cardiac magnetic resonance imaging
IVUS: Intravascular ultrasound
HF: Heart failure
re-AMI: Recurrence of AMI
IQR: Interquartile range
LAD: Left atrial diameter
ACEI: Angiotensin-converting enzyme inhibitors
ARB: Angiotensin receptor blockers
NDHP-CCB: Non-dihydropyridine calcium channel blockers
CAD: Coronary artery disease
FFR: Fractional flow reserve
